# Cluster analysis of sputum cytokine-high profiles reveals diversity in T(h)2-high asthma patients

**DOI:** 10.1186/s12931-017-0524-y

**Published:** 2017-02-23

**Authors:** Sven F. Seys, Hans Scheers, Paul Van den Brande, Gudrun Marijsse, Ellen Dilissen, Annelies Van Den Bergh, Pieter C. Goeminne, Peter W Hellings, Jan L. Ceuppens, Lieven J Dupont, Dominique M. A. Bullens

**Affiliations:** 1 Lab of clinical immunology, Department of Microbiology and Immunology, Herestraat 49/811, 3000 Leuven, KU Belgium; 2 Department of Public Health and Primary Care, Environmental Health Unit, Lab of pneumology, Leuven, KU Belgium; 3 Respiratory department, Leuven, UZ Belgium; 4 Lab of respiratory disease, and lab of pediatric immunology, Department of Clinical and Experimental Medicine, Leuven, KU Belgium; 5 ENT department, Leuven, UZ Belgium; 6 Paediatric department, Leuven, UZ Belgium; 7 Lab of paediatric immunology, Department of Microbiology and Immunology, Leuven, KU Belgium

**Keywords:** Airway inflammation, Type 2 inflammation, Endotype, Phenotype, Precision medicine

## Abstract

**Background:**

Asthma is characterized by a heterogeneous inflammatory profile and can be subdivided into T(h)2-high and T(h)2-low airway inflammation. Profiling of a broader panel of airway cytokines in large unselected patient cohorts is lacking.

**Methods:**

Patients (*n* = 205) were defined as being “cytokine-low/high” if sputum mRNA expression of a particular cytokine was outside the respective 10^th^/90^th^ percentile range of the control group (*n* = 80). Unsupervised hierarchical clustering was used to determine clusters based on sputum cytokine profiles.

**Results:**

Half of patients (*n* = 108; 52.6%) had a classical T(h)2-high (“IL-4-, IL-5- and/or IL-13-high”) sputum cytokine profile. Unsupervised cluster analysis revealed 5 clusters. Patients with an “IL-4- and/or IL-13-high” pattern surprisingly did not cluster but were equally distributed among the 5 clusters. Patients with an “IL-5-, IL-17A-/F- and IL-25- high” profile were restricted to cluster 1 (*n* = 24) with increased sputum eosinophil as well as neutrophil counts and poor lung function parameters at baseline and 2 years later. Four other clusters were identified: “IL-5-high or IL-10-high” (*n* = 16), “IL-6-high” (*n* = 8), “IL-22-high” (*n* = 25). Cluster 5 (*n* = 132) consists of patients without “cytokine-high” pattern or patients with only high IL-4 and/or IL-13.

**Conclusion:**

We identified 5 unique asthma molecular phenotypes by biological clustering. Type 2 cytokines cluster with non-type 2 cytokines in 4 out of 5 clusters. Unsupervised analysis thus not supports *a priori* type 2 versus non-type 2 molecular phenotypes. www.clinicaltrials.gov NCT01224938. Registered 18 October 2010.

**Electronic supplementary material:**

The online version of this article (doi:10.1186/s12931-017-0524-y) contains supplementary material, which is available to authorized users.

## Background

Chronic airway inflammation has long been seen as a general characteristic of all patients with asthma [1]. In addition, dyspnoea, wheezing and cough are typical asthma symptoms and result from narrowing of the airway lumen. Corticosteroids and bronchodilators are therefore the first choice treatment for patients with asthma [2]. The response to these drugs is variable, which could not only be attributed to pharmacogenetic aspects [3]. Analysis of inflammatory cells in sputum and bronchial biopsies revealed that eosinophilic inflammation was only present in a subgroup of patients and that its presence is associated with the beneficial response to corticosteroids [4]. Molecular phenotyping has further strengthened the concept of asthma as a heterogeneous disease [5]. Patients with a T(h)2-high profile are found in half of the mild asthmatic patients and reported to be responsive to steroids [6, 7]. Biomarker research, so far, has mainly focused on T(h)2 associated cytokines or surrogate markers of type 2 driven airway inflammation (such as F_E_NO or serum periostin). It remains unclear which pathophysiological mechanisms are driving asthma in patients with normal airway expression of type 2 cytokine levels.

Several other T cell related and epithelial derived cytokines are found to be increased in patients with asthma [8–11]. However, how they are associated which each other is not fully understood. Microarray analysis of sputum cells revealed 6 biomarkers that are specifically associated with eosinophilic or neutrophilic asthma [12]. A recent cluster analysis showed 6 clinicopathobiologic clusters with differences in lung function, sputum cellular profile, YKL-40 protein and matrixmetalloproteinases [13]. We previously showed that sputum analysis can be used to identify different “cytokine-high” patterns and that these patterns are linked to lung function parameters, asthma control and BMI [11, 14].

There is thus an increased awareness about the diversity of the inflammatory profile amongst asthma patients. Unravelling the associations between the different inflammatory cells and mediators might help to identify the patients’ endotype. In the present study we aimed to extent our previous findings in a larger population of asthmatics and identify cytokine-based asthma clusters in which each patient is allocated to one single cluster. To that aim, expression of twelve sputum cytokines was assessed and analysed by unsupervised hierarchical cluster analysis.

## Methods

### Subjects

Patients (*n* = 296) with symptoms compatible with asthma between 18 and 65 years were recruited amongst those consecutively attending the outpatient clinic of pulmonary disease or allergology of the University Hospital Leuven. Diagnosis of asthma was confirmed based on previous (<24 months) or current proof of reversibility of FEV_1_ ≥ 12% after inhalation of salbutamol and/or a positive histamine provocation test (PC_20_ < 8 mg/ml). Patients with respiratory infection 1 month or exacerbation 3 months prior to analysis were excluded. Patients were allowed to continue daily treatment. Patients who did not take inhaled steroids for at least 3 months were classified as steroid-naive patients. Healthy non-symptomatic volunteers (*n* = 96) were recruited amongst students and university coworkers. A power analysis was conducted to determine the number of asthma patients and healthy subjects needed to detect a 2-fold difference in the mean values between both groups given current knowledge on sputum cytokine expression levels, based on our own previously published results [8, 10]. Power analysis showed that 300 asthma patients and 100 healthy subjects were required, considering that 70% of individuals will produce a sample that is useful for cytokine analysis. Written informed consent was obtained from all patients. Study was approved by the local ethical committee and registered on clinicaltrials.gov (NCT01224938). Samples of 34 asthma patients and 20 control subjects in the current study were also previously used for analysis of sputum cytokine mRNA patterns in asthma [11]. However, samples were re-analyzed together with the enlarged cohort for sputum cytokine analysis.

### Lung function and clinical characteristics

Different dynamic lung volumes were measured by spirometry (Jaeger, Carefusion): FEV_1_ (forced expiratory volume in 1 second), FVC (forced vital capacity) and FEF_25–75_ (forced expiratory flow at 25–75% of FVC); and expressed as % predicted. FEV_1_ after 2 years was retrieved from medical records of patients in follow up (see Additional file 1). F_E_NO was measured prior to spirometry by means of a chemiluminescence analyzer (CLD88s, Ecomedics, Switzerland). Spirometry was performed according to ERS criteria, before and after inhalation of salbutamol 400 μg. Asthma control was assessed by Asthma Control Test questionnaire [15]. Atopy was assessed by skin prick test or immunocap (Phadia) for most common aeroallergens: house dust mite, grass pollen mixture, tree pollen mixture, cat, dog, Alternaria alternata, Aspergillus fumigatus (HAL Allergy, Leiden).

### Sputum induction and analysis

Sputum induction and processing was performed as described previously [10, 11, 16, 17]. In brief, hypertonic salt solution of 3, 4 and 5% respectively was nebulized each time 7 min. Afterwards, the patient was asked to rinse the mouth and spit the sputum in a collection tube. Sputum total and differential cell counts were obtained by cytospin (Shandon cytocentrifuge). An a priori selected panel of T cell and innate cytokines were analyzed by by real-time (RT)-PCR: Th1 (IFN-γ), Th2 (IL-4, IL-5, IL-13 and IL-10), Th17 (IL-17A, IL-17 F and IL-22) and innate (IL-1β, IL-6, IL-25, and TNF). Patients were defined as being “cytokine-low” or “cytokine-high” if sputum mRNA expression levels of that particular cytokine were outside the 10^th^–90^th^ percentile value of the current control group, respectively. Samples with an mRNA content of <0.25 μg and <300 000 non-squamous sputum cells were excluded. We measured cytokine mRNA levels only in samples with β-actin mRNA levels >10 000 copies. Cytokine mRNA levels were measurable in 70% (205/296 patients) of all included asthma patients and 83% (80/96 subjects) of healthy subjects (see Additional file 1: Table E1).

### Cluster analysis and statistics

A tertiary code (1: “cytokine-high”, −1: “cytokine-low” or 0: normal cytokine levels for a particular cytokine) was created and used for unsupervised hierarchical cluster analysis. Ward’s minimum-variance clustering method was used to create the best set of clusters for each possible number of clusters, and we decided upon the number of clusters to proceed by combining the cubic clustering criterion and pseudo F and T^2^ statistics [18, 19]. A tree representing the patients grouped in clusters was created. Cluster analysis was performed in SAS, version 9.3 (SAS Institute, Cary, NC, USA).

Further statistical analyses were performed with Graphpad Prism V for Macintosh (Graphpad Software Inc., San Diego, USA) by use of Kruskal-Wallis, Dunn’s or Tukey multiple comparison test (as post-test) and Mann–Whitney *U* test when appropriate. ANOVA and *T*-test were performed if data were normally distributed. Chi squared analysis was used to compare proportions between different groups. Normality was analyzed by Kolmogorov-Smirnov test. Mean or median levels of clinical and inflammatory parameters in different clusters were compared to the actual mean or median of the total group of asthmatics. For multivariate analysis of factors that may contribute to cluster determination, multinomial logistic regression analysis was applied. A difference was considered to be significant when *p* < 0.05.

## Results

### Subjects

Subject characteristics are presented in Table 1. Sensitisation to aeroallergens was more prevalent amongst asthmatic patients (76%) than controls (35%) (Table 1). Active smoking was recorded in 25 asthmatics (12%). BMI was higher in asthmatics compared to controls (*p* < 0.0001). Twenty percent of patients (*n* = 41) were steroid-naive. Fifty-three percent of asthmatics had a Th2-high profile (sputum IL-4-, IL-5- and/or IL-13-high).

**Table 1:** Subject characteristics

	Asthma (*n* = 205)	Healthy (*n* = 80)	*P* value
Subjects (n=)	205	80	
Age (years)	30–40–51	23–27–42	<0.0001
Gender (M/F)	96/112	30/50	0.09
Body Mass Index	21.9–24.4–28.1	20.4–22.3–24.5	<0.0001
Active smoking (%)	25 (12%)	2 (3%)	0.007
Allergy (%)	139 (76%)	23 (35%)	<0.0001
Steroid-naive	41 (20%)	NA	
Inhaled steroids only	142 (69%)	NA	
Inhaled and oral steroids	22 (11%)	NA	
FEV_1_ % predicted (%)	94.6 ± 19.0	108.9 ± 14.2	<0.0001
FEV_1_/FVC (%)	72.3 ± 11.4	81.7 ± 6.5	<0.0001
F_E_NO (ppb)	13.4–22.0–38.6	10.3–16.2–22.9	0.0004
Sputum eosinophils (%)	0.0–1.0–4.0	0.0–0.0–0.4	<0.0001
Sputum neutrophils (%)	13.0–35.0–58.8	13.8–25.4–41.1	0.02
Sputum total cell count (x10^6^ cells)	0.8–1.3–2.5	0.9–1.4–2.0	0.48
Asthma Control test (/25)	17.0–20.5–23.0	25–25–25	<0.0001

Clinical, lung function and inflammatory parameters in asthmatic patients and healthy subjects. Normally distributed data were represented as mean ± standard deviation and analyzed by *T*-test. Data that were not normally distributed were represented as median and 25–75% (interquartile range) percentile and analyzed by Mann-Whitney test. *FEV*_*1*_ Forced Expiratory Volume in 1 second, *FVC* Forced Vital Capacity, *F*_*E*_ *NO* Fraction of exhaled Nitric Oxide

### Sputum cytokine profiles in asthma patients: cluster analysis

**Fig. 1:**
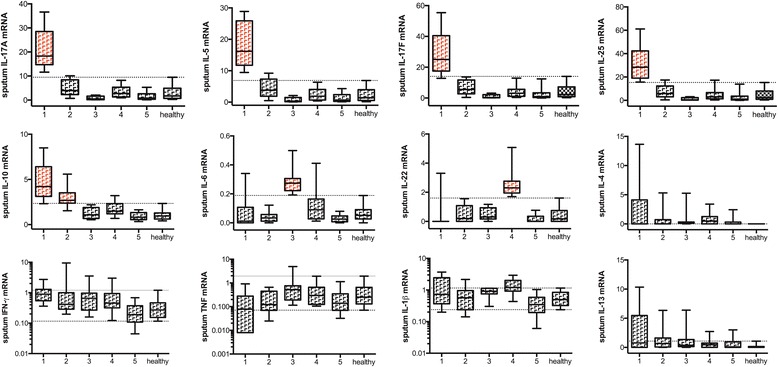
Absolute sputum cytokine levels among different clusters of asthmatics. Patients were clustered based on their sputum cytokine-high or cytokine-low profile. Asthmatics are divided into 5 clusters: cluster 1: *n* = 24, IL-5-high and IL-17 F-high; cluster 2: *n* = 15, IL-5-high and IL-17 F-low; cluster 3: *n* = 8, IL-6-high; cluster 25: *n* = 15, IL-22-high; cluster 5: *n* = 132. Absolute sputum cytokine levels were shown as 10-90^th^ percentile box and whiskers plots. The dotted line represents the 10^th^ or 90^th^ percentile value of control individuals

Sputum cytokine mRNA 10^th^ and 90^th^ percentile values of expression levels in healthy subjects (*n* = 80) were calculated to determine lower and upper cut-off levels for the various cytokines (Table 2). Unsupervised hierarchical clustering was applied to identify clusters of patients with a similar sputum “cytokine-low or -high” profile. Five clusters were selected according to cubic clustering criterion (CCC) and pseudo F and T^2^ statistics (see Additional file 1: Figure E1A). Patients in the first cluster (*n* = 24) presented with an “IL-5-, IL-10-, IL-25-, IL-17A- and IL-17 F-high” profile. Patients in the second cluster (*n* = 16) presented with an “IL-5- and/or IL-10-high” profile but normal IL-17 F levels. Patients in cluster 3 (*n* = 8) had an “IL-6-high” profile. Patients in cluster 4 (*n* = 25) presented with an “IL-22-high” profile of which half were also “IL-1β-high”. Cluster 5 (*n* = 132) was the largest cluster and consists of patients with normal levels of the former cytokines (*n* = 123). The proportion of patients with an “IL-4- or IL-13-high” profile was equally distributed among the 5 clusters. Absolute sputum cytokine levels among the 5 clusters are shown in Fig. 1. Patients with a “TNF-low” profile were significantly overrepresented in cluster 1 and 5 compared to the other clusters (*p* = 0.01), whereas those with an “IL-1β-low” profile were significantly overrepresented in cluster 5 (*p* < 0.0001).

**Table 2:** Sputum cytokine mRNA levels in control and asthmatic individuals

	Control (*n* = 80)	Asthma (*n* = 205)	*P* value	Adjusted *P* value*	“Cytokine-high” *n*=, (%)	“Cytokine-low” *n*=, (%)
IFN-γ	0.12 – 0.27 – 1.20	0.07 – 0.30 – 1.26	0.35	0.42	22 (11%)	38 (19%)
IL-4	0.00 – 0.00 – 0.00	0.00 – 0.00 – 3.14	<0.0001	0.0006	81 (40%)	0
IL-5	0.16 – 1.41 – 6.89	0.15 – 1.36 – 10.3	0.49	0.49	30 (15%)	22 (11%)
IL-13	0.00 – 0.00 – 1.07	0.00 – 0.27 – 4.10	<0.0001	0.0006	56 (27%)	0
IL-10	0.42 – 0.96 – 2.34	0.44 – 1.20 – 3.52	0.018	0.04	41 (20%)	19 (9%)
IL-17A	0.27 – 1.73 – 9.49	0.21 – 1.62 – 12.55	0.26	0.35	26 (13%)	33 (16%)
IL-17 F	0.25 – 2.3 – 14.02	0.16 – 1.99 – 16.41	0.15	0.3	23 (11%)	34 (17%)
IL-22	0.00 – 0.17 – 1.60	0.00 – 0.14 – 2.06	0.49	0.49	29 (14%)	0
IL-25	0.34 – 2.37 – 15.28	0.19 – 2.18 – 17.78	<0.0001	0.0006	26 (13%)	34 (17%)
TNF	0.07 – 0.25 – 1.95	0.03 – 0.14 – 1.95	0.22	0.35	7 (3%)	49 (24%)
IL-6	0.00 – 0.05 – 0.19	0.00 – 0.03 – 0.17	0.0002	0.0006	16 (8%)	28 (14%)
IL-1β	0.24 – 0.50 – 1.15	0.08 – 0.48 – 1.68	0.25	0.35	35 (17%)	51 (25%)

Sputum cytokine mRNA levels were represented as median and 10^th^–90^th^ percentile value and analysis between both groups was performed by Mann-Whitney test. *P value after correction for multiple comparison (Benjamini-Hochberg correction)

Validation of the number of clusters and the patients’ cytokine profile in each cluster was performed by splitting the total cohort into 2 groups and repeating the analysis on each half separately. Analysis of the estimated number of cluster (by Ward’s method) showed 5 clusters for the first group and 6 for the second group. Cluster analysis was performed for 5 clusters for sake of consistency in both groups. To compare the cytokine-high profiles among the different clusters, radar graphs were used, representation the proportion of patients with a particular cytokine-high expression profile (Additional file 1: Figure E2). The five clusters were remarkably similar in both analyses, except for the IL-22-high cluster, which could not be found in one of the groups. If for one of the groups 6 clusters were build (as suggested by Ward’s method) then all five main clusters remained and only 1 patient was separated from the cluster with 77 patients.

### Evaluation of lung function and airway inflammation

**Fig. 2:**
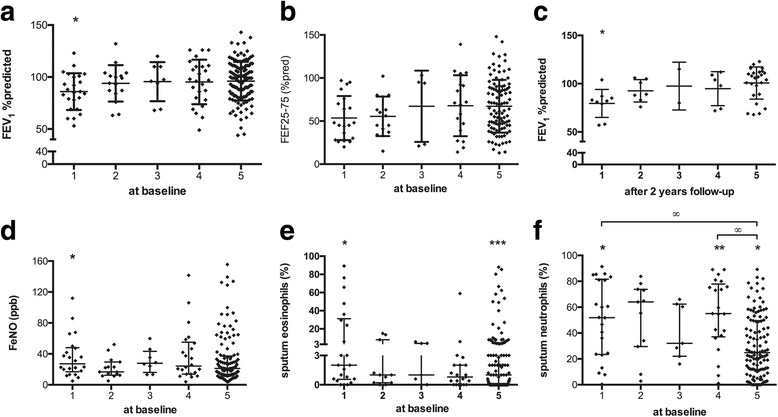
Lung function and airway inflammatory parameters. Asthmatics are divided into 5 clusters: cluster 1: *n* = 24, IL-5-high and IL-17 F-high; cluster 2: *n* = 15, IL-5-high and IL-17 F-low; cluster 3: *n* = 8, IL-6-high; cluster 25: *n* = 15, IL-22-high; cluster 5: *n* = 132. Data are represented as mean ± standard deviation (**a**-**c**) or median ± interquartile range (**d**-**f**). Data are compared between the 5 clusters by Kruskal-Wallis and Dunn’s Multiple comparison test (∞: *p* < 0.05). Data of each cluster was compared to the mean (**a**-**c**) or median (**d**-**f**) of the total group (*: *p* < 0.05, **:*p* < 0.01; ***:*p* < 0.001)

The group with an “IL-5-, IL-10-, IL-17A/F-, IL-25-high” profile (cluster 1) had significantly lower FEV_1_ % predicted compared to the mean of all asthmatics (*p* = 0.026; Fig. 2a and Table 3). Patients in cluster 1 also had significantly higher chance to have a FEV_1_ % predicted ≤85% (OR: 2.7, 95% CI: 1.1-6.4). A similar trend was found for FEF_25–75_, % predicted levels in cluster 1 (*p* = 0.079; Fig. 2b).

**Table 3:** Characteristics of patient clusters

CLUSTER	1	2	3	4	5	*P* value
Subjects (n=)	24	16	8	25	132	
Age (years)	44.0 ± 13.4	47.3 ± 14.1	39.0 ± 15.4	38.0 ± 12.7	40.2 ± 12.7	0.14
Gender (M/F)	12/12	6/10	4/4	11/14	62/70	0.95
Body Mass Index	22.5–26.8–28.8	21.2–25.4–29.9	20.7–23.5–30.9	21.9–23.6–26.0	21.8–24.3–28.7	0.55
Active smoking (%)	9	0	0	19	19	0.27
Atopy (%)	73	62	88	73	80	0.54
Steroid-naive (%)	8	18	12	20	23	0.82
Inhaled steroids only (%)	79	75	50	68	68	0.58
Inhaled and oral steroids (%)	13	7	38	12	9	0.15
FEV_1_, % predicted	86.0 ± 17.7^†^	93.9 ± 17.5	95.6 ± 18.8	95.2 ± 21.4	96.1 ± 18.7	0.21
FEV_1_/FVC	69.0 ± 12.0	72.5 ± 8.1	71.3 ± 13.1	71.3 ± 13.4	73.1 ± 11.3	0.57
PEF, % predicted	90.0 ± 19.7	91.8 ± 18.5	96.9 ± 26.6	88.8 ± 26.7	97.5 ± 19.5	0.30
FEF_25–75_, % predicted	53.5 ± 25.6	55.5 ± 23.0	67.2 ± 41.4	67.8 ± 35.4	67.2 ± 30.6	0.29
F_E_NO (ppb)	16.8–27.2–48.1^†^	12.1–6.7–29.5	16.1–27.9–43.3	13.9–24.3–55.1	13.3–21.2–37.2	0.46
Sputum eosinophils (%)	0.6–2.0–31.1^†^	0.2–1.0–8.0	0.0–1.0–4.0	0.0–0.8–2.0	0.0–1.0–4.0^†††^	0.36
Sputum neutrophils (%)	23.5–51.9–81.6^*,†^	29.5–64.0–73.8	22.0–32.0–62.4	37.0–55.0–77.9^**,††^	11.8–24.8–49.5^†^	<0.0001

Clinical, lung function and inflammatory parameters in different asthma patients clusters defined by their sputum cytokine profile. Cluster 1: *n* = 24; IL-5-high and IL-17A-high, cluster 2: *n* = 16; IL-5-high and IL-10-high and IL-17 F-low, cluster 8: *n* = 15; IL-6-high, cluster 4: *n* = 25; IL-22-high, cluster 5: *n* = 132; normal levels of the previous cytokines with or without an IL-1β or TNF low profile. Normally distributed data were represented as mean ± standard deviation and analyzed by ANOVA. Data that were not normally distributed were represented as median and 25–75% (interquartile range) percentile and analyzed by Kruskal-Wallis test. Dunn’s Multiple Comparison test was used as a post hoc test. ^*,**^ *p* < 0.05, *p* < 0.01 respectively compared to cluster 5, ^†,††,†††^ *p* < 0.05, *p* < 0.01 and *p* < 0.001 respectively compared to mean/median level in asthmatics. *FEV*_*1*_ Forced Expiratory Volume in 1 second, *FVC* Forced Vital Capacity, *PEF* Peak Expiratory Flow, *FEF*_*25–75%*_ Forced expiratory Flow at 25–75% interval, *F*_*E*_ *NO* Fraction of exhaled Nitric Oxide

After 2 and 3 years (*p* < 0.05), FEV_1_ % predicted was significantly lower in cluster 1 compared to the mean of all asthmatics (Fig. 2c and see Additional file 1: Figure E3).

F_E_NO levels of patients in cluster 1 were significantly higher compared to the median of all asthmatics (*p* = 0.044; Fig. 2f). Sputum eosinophil percentages in parallel were significantly higher in cluster 1 but also in cluster 5 compared to the median of all asthmatics (*p* = 0.01 and *p* = 0.003; Fig. 2d and Table 3). Sputum neutrophil percentages were significantly higher in cluster 1 and 4 (*p* = 0.039 and *p* = 0.007; Fig. 2e) and significantly lower in cluster 5 (*p* = 0.018; Fig. 2e) compared to the median of all asthmatics. Both cluster 1 and 4 had significantly higher sputum neutrophil percentages compared to cluster 5 (*p* < 0.05 and *p* < 0.01; Fig. 2e). A schematic representation of the clusters based on their sputum eosinophil and neutrophil profile is visualized in Additional file 1: Figure E4.

### Patient cluster decision tree

**Fig. 3:**
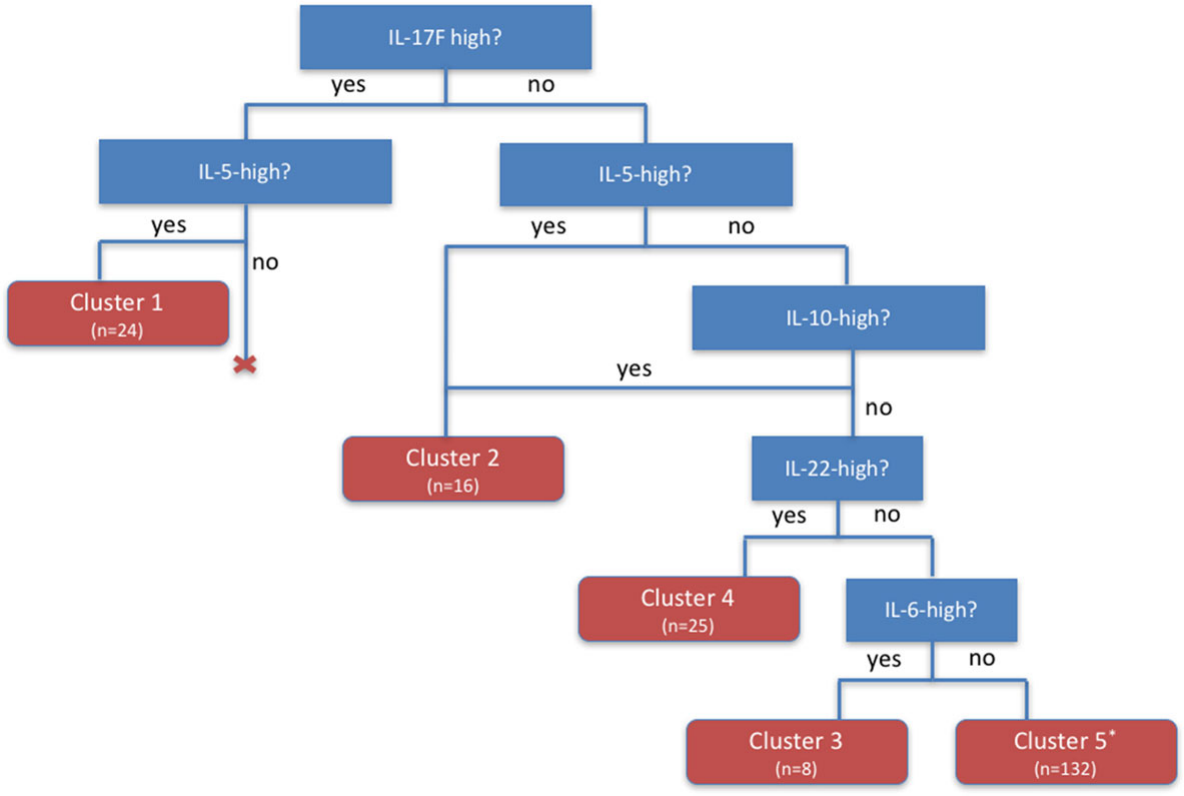
Decision tree with patient clusters of all asthmatics. Patients with an “IL-17 F-high and IL5-high” profile, irrespective of expression of other cytokines, were labelled as cluster 1 (*n* = 24). Patients with an “IL-5-high or IL-10-high” but not an “IL-17 F-high” profile, were identified as cluster 2 (*n* = 16). In the next step, patients who had normal levels of the previous cytokines but were “IL-22-high”, were assigned to cluster 3 (*n* = 25). Patients with an “IL-6-high profile” were labelled as cluster 4 (*n* = 8). All other patients were grouped in cluster 5 (*n* = 132). *: This group consists of patients with normal levels of the previous cytokines with or without an “IL-1β- or TNF-low” profile (*n* = 49 and *n* = 83, respectively)

A discriminative decision tree to classify all asthma patients individually into the cited clusters was developed afterwards (Fig. 3). Patients with an “IL-5- and IL-17 F-high” profile, irrespective of expression of other cytokines, were classified in cluster 1 (*n* = 24). Patients with an “IL-5- and/or IL-10-high” but not an “IL-17 F-high” profile, were classified in cluster 2 (*n* = 16). In the next step, patients who have normal levels for the previous cytokines but “IL-22-high”, were assigned to cluster 4 (*n* = 25). Next, patients with an “IL-6-high profile”, are classified in cluster 3 (*n* = 8). All other patients were grouped in cluster 5 (*n* = 132). This group consists of patients with normal levels of the previous cytokines. By use of this decision tree, all patients could be classified in one single cluster without overlap.

### Sputum cytokine profiles in steroid-naive asthma patients

**Fig. 4:**
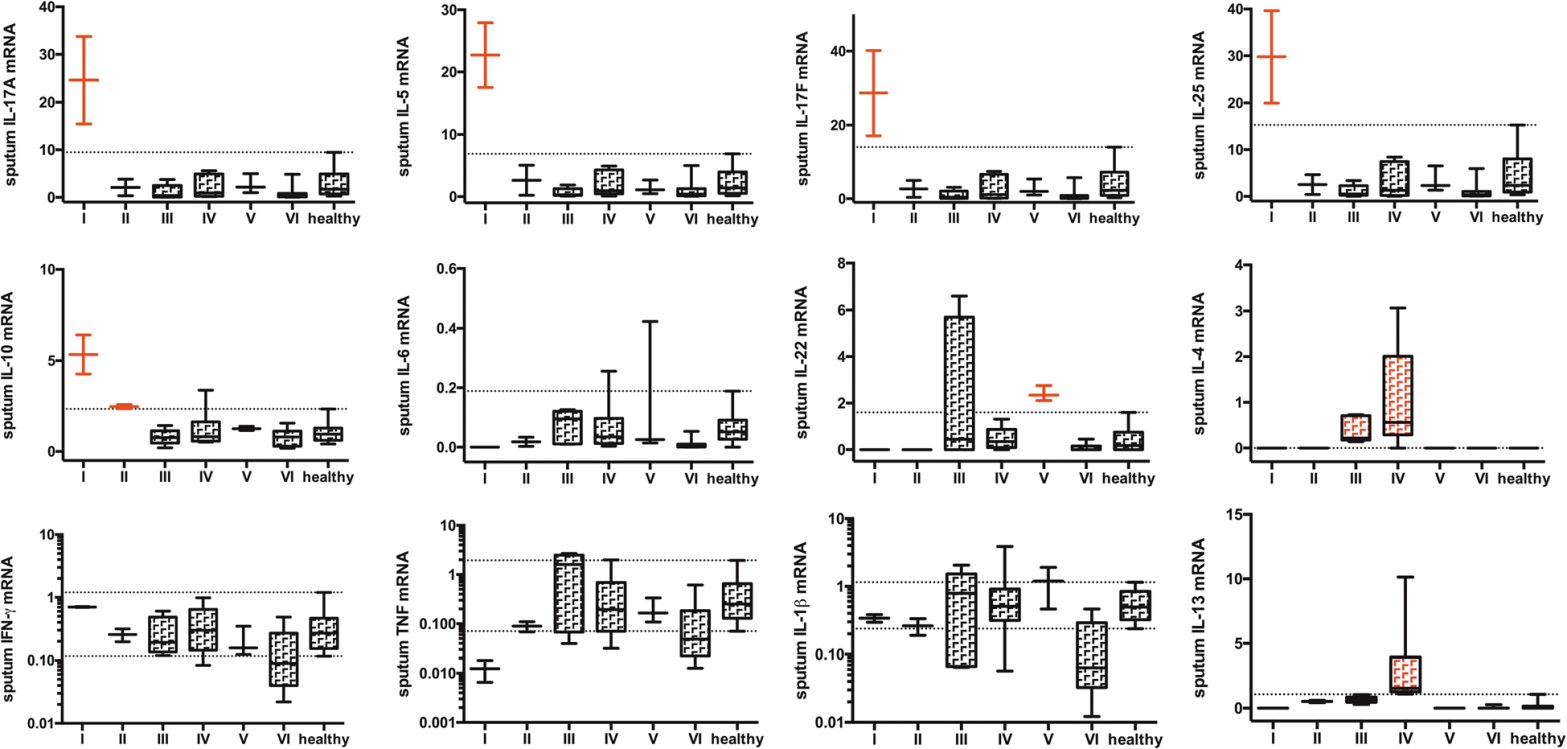
Absolute sputum cytokine levels among different clusters of steroid-naive asthmatics. Patients were clustered based on their sputum cytokine-high or cytokine-low profile. Absolute sputum cytokine levels were shown as 10–90^th^ percentile box and whiskers plots. The dotted line represents the 10^th^ or 90^th^ percentile value of control individuals

Forty-one patients did not use inhaled steroids daily. Patient characteristics are shown in Additional file 1: Table E1. Among them, six clusters (according to CCC, pseudo F and t^2^ statistics; Additional file 1: Figure E1B) could be identified: cluster I (similar profile to cluster 1) “IL-5-, IL-25-, IL-17A-, IL-17 F- and IL-10-high” (*n* = 2), cluster II (similar to cluster 2) “IL-5- and/or IL-10-high” but “IL-17 F-low” (*n* = 2), cluster III “IL-4-high” (*n* = 5), cluster IV “IL-4- and IL-13-high” (*n* = 9), cluster V (similar profile to cluster 4) “IL-22-high” (*n* = 3) and cluster VI (similar profile to cluster 5) normal cytokine levels or “IL-1β- or TNF-low” (*n* = 20). Patients with an “IL-6-high” profile (*n* = 2) did not cluster and were found in cluster IV and V. Absolute sputum cytokine mRNA levels among the different clusters were shown in Fig. 4.

**Fig. 5:**
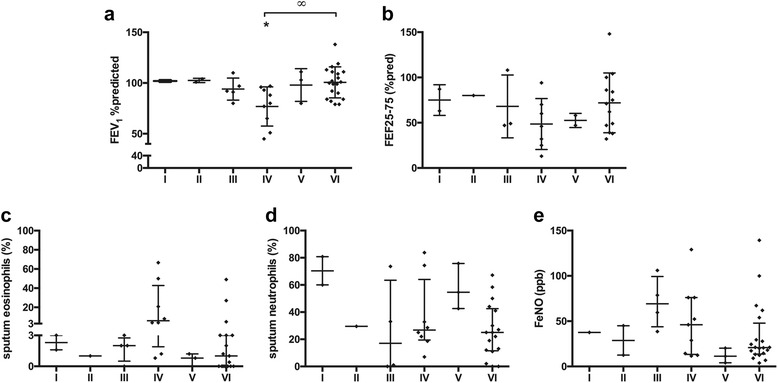
Lung function and airway inflammatory parameters of steroid-naive asthmatics. Steroid-naive asthmatics are divided into 6 clusters: cluster I: *n* = 2, IL-5-high and IL-17 F-high; cluster II: *n* = 2, IL-5-high or IL-10-high and IL-17 F-low; cluster III: *n* = 5, IL-4-high; cluster IV: *n* = 10, IL-4-high and IL-13-high; cluster V: *n* = 3, IL-22-high; cluster VI: *n* = 20. Data are represented as mean ± standard deviation (**a**-**b**) or median ± interquartile range (**c**-**e**). FEV_1_% predicted levels of each cluster were compared to the mean of the total group (*: *p* < 0.05)

Among steroid-naive patients, FEV1 % predicted was significantly lower in those with an “IL-4- and IL-13-high” profile (cluster IV) compared to the mean of all asthmatics (*p* = 0.025; Fig. 5a and Additional file 1: Table E1).

Sputum eosinophil percentages were significantly increased in patients with an “IL-4- and IL-13-high” profile compared to the median of all asthmatics (*p* = 0.04; Fig. 5c), while no significant differences were found for sputum neutrophils (Fig. 5d). F_E_NO levels did not significantly differ among the 6 clusters but were highest in cluster III and IV (Fig. 5e).

### Multivariate analysis of confounding factors

In order to assess whether confounding factors such as age, gender, BMI and steroid use may have influenced the classification of the patient clusters, these factors were studied by multinomial logistic regression analysis, both separately in univariate analyses and together in a multivariate model. None of the factors included in the univariate analysis contributed to the classification of the patient clusters (data not shown). Multivariate logistic regression did not suggest an association either between any of these factors and the identified clusters (Additional file 1: Table E3; *p* = 0.52).

## Discussion

We have identified 5 patient clusters based on sputum cytokine-high profiles in a large unselected asthma cohort. Remarkably, type 2 cytokines, IL-4 and IL-13, did not cluster but were found in an equal proportion of patients throughout the 5 clusters. This points towards considerable heterogeneity amongst patients with type 2 inflammation. Many patients (cluster 1–4) show additional inflammation on top of IL-4 and IL-13. That might be the reason why patients with an “IL-4- and/or IL-13-high” profile do not show up as a separate cluster. In contrast, steroid-naive patients can either have milder or more recently developed disease. This could explain why they might present with limited additional cytokine-high expression on top of their IL-4-/IL-13-high expression. Hence, steroid-naive patients with an “IL-4- and/or IL-13-high” profile cluster together.

Patients with high IL-5 mRNA expression, on the contrary, were restricted to cluster 1 (*n* = 24) and expressed high IL-17 F mRNA levels. Patients in this cluster also have an “IL-17A-, IL-25- and IL-10-high” profile, but in contrast to IL-5 and IL-17 F, these cytokines were not required for identification of the cluster. The identification of this cluster is in agreement with our earlier observation that sputum IL-5, IL-25 and IL-17A mRNA levels are increased simultaneously in a subgroup of asthmatics [11]. At first, this association was unexpected, as we hypothesized that IL-5 would have been increased in eosinophilic asthma, whereas IL-17A might be increased in neutrophilic asthma. Recently, Hinks and colleagues also demonstrated an association between BAL IL-17A and eosinophil counts in asthmatics [20]. Patients in cluster 1 furthermore had increased sputum eosinophils as well as neutrophils, matching with the IL-5- and IL-17-high cytokine profile. These patients had worse lung function parameters and 2.7 higher odds to have FEV_1_ ≤ 85%. Analysis of lung function parameters after 2 and 3 years still showed worst FEV_1_ suggesting persistence of airway obstruction in these patients. A prospective longitudinal study however should help to define the lung function trajectory of those patients.

Patients in cluster 2 had high IL-10 and some had high IL-5 (as in cluster 1) but normal IL-17A/F mRNA levels, and these patients had slightly better lung function parameters compared to cluster 1, indicating that high IL-17 F expression associates with worse lung function parameters. Patients in cluster 3 had high IL-6 sputum mRNA levels, whereas cluster 4 is characterized by high IL-1β and IL-22 mRNA. These clusters represent patients with normal sputum eosinophils in most cases, whereas neutrophils were increased in patients with high IL-1β and IL-22. Single nucleotide polymorphisms in the IL-6 receptor gene were shown to be associated with increased risk for asthma [21]. Patients in cluster 3 might therefore be good candidates for trials with anti-IL6 monoclonal antibodies. Strikingly, IL-22 identified a separate patient cluster (cluster 4) and was not increased in patients with high IL-17A/F (cluster 1). This was unexpected as IL-22 is thought to be produced by Th17 cells, which we consider responsible for IL-17A/F production in this cluster. Different other cells may however also produce IL-22: alveolar macrophages, dendritic cells, Th22 cells and innate lymphoid cells [22–24]. Th22 cells not producing IL-17A could be identified recently [25]. IL-22 expression was reported to be increased in peripheral blood mononuclear cells (PBMC) from both children and adults with asthma [26, 27]. In another study, IL-22 attenuated IL-25 production by lung epithelial cells and inhibited antigen-induced eosinophilic airway inflammation, underscoring that IL-22 might exert protective effects in asthma [28]. However, in contrast to the idea that IL-22 might be beneficial in asthma, analysis of patients in cluster 4 did not show significantly better lung function than the other groups. Those patients had rather high sputum neutrophils and normal sputum eosinophil counts.

A heterogeneous group of patients was found in cluster 5. More than half of the patients had no increased cytokine expression or sputum granulocytes. A low degree of inflammation is thus found in these patients, which may correspond to the paucigranulocytic phenotype [29]. In contrast, 40% of patients had an “IL-4- and/or IL-13-high” profile, which is equal to the proportion found in the other clusters. However, in this cluster, this occurs without elevation of any other T helper or pro-inflammatory cytokines, which discriminates them from the other clusters. Cluster 5 had rather low levels of IFN-γ, TNF and IL-1β in sputum. Sputum eosinophils were increased in a subgroup of patients within cluster 5. This could be attributed to patients with an “IL-4- and/or IL-13-high” profile, as these patients have significantly higher sputum eosinophils and F_E_NO levels compared to patients with an normal levels of IL-4 and/or IL-13 profile (data not shown). In a study by Fahy and coworkers, a qPCR-based metric combining sputum cytokine expression of IL-4, IL-5 and IL-13 was used to distinguish between T(h)2-high and T(h)2-low asthmatics [7, 30]. In our hands, co-expression of all three cytokines is only found in a limited proportion of patients located in cluster 1.

The predominant cytokine-high profile in steroid-naive patients was “IL-4- and/or IL-13high”. In contrast to steroid-treated patients, expression of most of the other cytokines was equal to that found in controls. Typical T(h)2 driven inflammation with high IL-4 and high IL-13 expression was associated with high sputum eosinophils. These patients might be the group of patients that will be responsive to therapy with inhaled steroids. On the other hand, high IL-5 expression is associated with high IL-17A/F expression and is linked to high sputum eosinophils and neutrophils, which might be refractory to inhaled steroids. This however still has to be proven.

Patients who are less symptomatic or less severe patients are not always able to expectorate sputum upon nebulization with hypertonic saline. This might have led to an underestimation of patients with a paucigranulocytic phenotype or normal cytokine expression in sputum. In addition, longitudinal follow up of patients is required to evaluate reproducibility and responsiveness to treatment of sputum cytokine expression.

Cytokine profiling of airway samples might help in future to decide upon the biological treatment that fits best for each individual patient [31, 32]. Determination of a panel of cytokines will be required since absence or presence of type 2 inflammation will not show whether to give anti-IL-5, anti-IL-4, anti-IL-13 or others. Moreover, since several cytokines might be elevated simultaneously in the same patient, blocking shared cytokine receptors (e.g.: IL-4Rα for IL-4 and IL-13 or IL-17RA for IL-17A, IL-17 F and IL-25) or pathways might be a better strategy than monoclonal antibodies that block only 1 cytokine.

## Conclusion

We here defined 5 asthma patient clusters based on sputum cytokine expression, which underscores the heterogeneity in airway inflammation among different asthma patients. When unsupervised cluster analysis is applied, a priori classification of type 2 versus non-type 2 molecular phenotypes does not show up. Determination of eosinophilic inflammation might not be sufficient and needs to be extended with detailed analysis of the sputum cytokine profile in order to decide upon the right anti-cytokine treatment.

## Additional file

Additional file 1: Cluster analysis of sputum cytokine-high profiles reveals diversity in T(h)2-high asthma patients. (ZIP 247 kb)

## Abbreviations

FEF25–75%: Forced expiratory volume at 25–75% of FVC
FENO: Fraction of exhaled nitric oxide
FEV1: Forced expiratory volume in 1 second
FVC: Forced vital capacity
IL: Interleukin
PC20: Provocative concentration at which a drop in FEV1 of 20% occurred
Th: T helper cell
