# Supplementary material for: Cluster analysis of sputum cytokine-high profiles reveals diversity in T(h)2-high asthma patients
Source: Respir Res. 2017 Feb 23;18:39. doi: 10.1186/s12931-017-0524-y (PMC5324270; doi:10.1186/s12931-017-0524-y)
Supplement: Additional file 1: — Cluster analysis of sputum cytokine-high profiles reveals diversity in T(h)2-high asthma patients. (ZIP 247 kb) [file 12931_2017_524_MOESM1_ESM.zip › art clusters (submitted) additional files - Resp Res-R1.docx]

**Additional file**

**Title**: Cluster analysis of sputum cytokine-high profiles reveals diversity in T(h)2-high asthma patients

**Additional methods**

LUNG FUNCTION

FEV_1_ % predicted was also analysed after 1 (n=67), 2 (n=50) and 3 (n=32) years in those patients who attended the outpatient clinic for follow up during the period of 1, 2 and 3 years ± 3 months.

PRIMER AND PROBE SEQUENCES

The primers and probes for β-actin, IL-4, IL-5, IL-6, IL-10, IL-17A, IL-22, IL-25, IFN-γ and TNF have been reported (1-3). All primers and probes were purchased from Applied Biosystems or Eurogentec S.A. The primer and probe sequence for IL-1β, IL-13 and IL-17F were designed with Primer Express (Applied Biosystems):

IL-1β FW 5’ ttg ctc aag tgt ctg aag cag c 3’

IL-1β RV 5’ caa gtc atc ctc att gcc act g 3’

IL-1β TP 5’ tac ctg agc tcg cca gtg aaa tga tgg 3’

IL-13 FW 5’ cag aac cag aag gct ccg c 3’

IL-13 RV 5’ cgt tga tca ggg att cca gg 3’

IL-13 TP 5’ tgc aat ggc agc atg gta tgg agc 3’

IL-17F FW 5’ tac att cac aga aag agc ttc ctg c 3’

IL-17F RV 5’ agt act tga cca tgg ctg ggc 3’

IL-17F TP 5’ caa agt aag cca cca gcg caa cat ga 3’

**Table E1. Subject recruitment and sputum analysis**


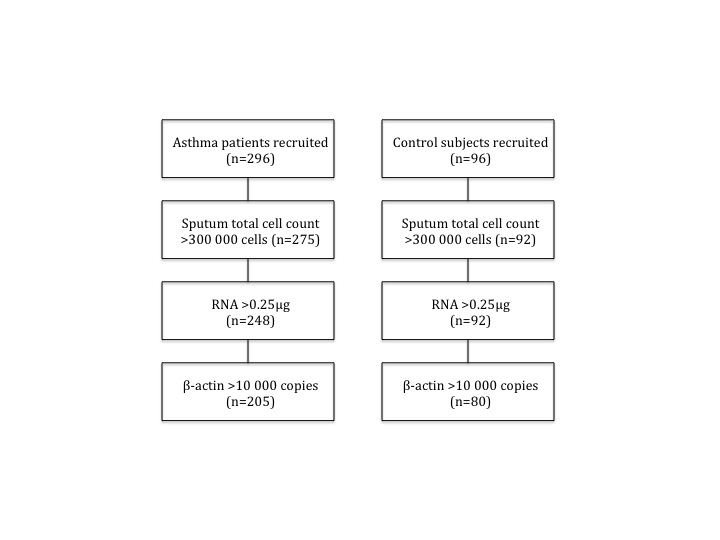


Samples with mRNA content of <0.25 μg and <300 000 sputum cells were excluded. We measured cytokine mRNA levels in samples with β-actin mRNA levels >10 000 copies. Cytokine mRNA levels were measurable in 70% (205/296 patients) of all included asthma patients and 83% (80/96 subjects) of healthy subjects.

**Table E2. Characteristics of steroid-naive patient clusters**

| CLUSTER | I | II | III | IV | V | VI | P value |
| --- | --- | --- | --- | --- | --- | --- | --- |
|  |  |  |  |  |  |  |  |
| Subjects (n=) | 2 | 2 | 5 | 9 | 3 | 20 |  |
| Age (years) | 31.5 ± 10.6 | 58.8 ± 6.4 | 36.4 ± 11.0 | 38.1± 11.9 | 41.7 ± 11.9 | 41.3 ± 14.3 | 0.40 |
| Gender (M/F) | 0/2 | 1/1 | 2/3 | 5/4 | 2/1 | 13/7 | 0.57 |
| Body Mass Index | 17.6-22.9-28.1 | 21.9-29.1-36.2 | 20.7-21.9-28.5 | 20.9-23.2-25.1 | 22.9-26.0-35.6 | 21.7-24.1-29.1 | 0.85 |
| Smoking (%) | 0 | 0 | 20 | 11 | 66 | 33 | 0.40 |
| Allergy (%) | 100 | 100 | 100 | 100 | 66 | 10 | 0.56 |
| FEV_1_, % predicted | 102.0 ± 1.4 | 102.5 ± 2.1 | 94.0 ± 10.9 | 76.9 ± 19.3^†, **^ | 98.0 ± 16.1 | 100.7 ± 15.3 | 0.018 |
| FEV_1_/FVC | 78.7 ± 13.7 | 74.9 ± 8.4 | 72.8 ± 10.3 | 67.8 ± 16.7 | 71.7 ± 2.5 | 75.1 ± 8.5 | 0.65 |
| PEF, % predicted | 101.5 ± 2.1 | 103.0 | 100.3 ± 10.3 | 81.57 ± 32.2 | 102.0 ± 14.1 | 101.1 ± 17.0 | 0.36 |
| FEF_25-75_, % predicted | 75.0 ± 17.0 | 80.0 | 68.0 ± 34.7 | 48.6 ± 28.2 | 52.5 ± 7.8 | 71.9 ± 33.0 | 0.53 |
| F_E_NO (ppb) | 37.5 | 12.5-44.9 | 43.8-69.2-99.3 | 13.4-46.0-76.1 | 4.0-11.3-20.2 | 13.6-20.8-47.8 | 0.17 |
| Sputum eosinophils (%) | 1.6-3.0 | 1.0 | 0.5-2.0-2.8 | 1.9-6.0-42.7 | 0.0-1.0-3.0 | 0.0-1.0-3.0 | 0.21 |
| Sputum neutrophils (%) | 60.0-80.8 | 29.5 | 0.3-17.0-63.5 | 19.5-26.8-64.0 | 42.6-54.6-75.8 | 11.5-25.0-42.5 | 0.06 |
|  |  |  |  |  |  |  |  |

Comparison of clinical, lung function and inflammatory parameters in different patients clusters of asthma defined by their sputum cytokine profile. Cluster I: n=2; IL-5-high and IL-17A-high, cluster II: n=2; IL-10-high and IL-5-low, cluster III: n=5; IL-4-high, cluster IV: n=9; IL-4- and/or IL-13-high, cluster V: n=3; IL-22-high; cluster VI: n=20; normal levels of the previous cytokines with or without an IL-1β or TNF low profile. Normally distributed data were represented as mean ± standard deviation and analyzed by ANOVA. Data that were not normally distributed were represented as median and 25-75% (interquartile range) percentile and analyzed by Kruskal-Wallis test. Dunn’s Multiple Comparison test was used as a post hoc test. ^**^: p<0.001 compared to cluster VI, ^†^: p<0.05 compared to mean level in asthmatics. FEV_1_: Forced Expiratory Volume in one second, FVC: Forced Vital Capacity, PEF: Peak Expiratory Flow, FEF_25-75%_: Forced expiratory Flow at 25-75% interval, F_E_NO: Fraction of exhaled Nitric Oxide.

Table E3. Multivariate analysis of demographic characteristics

| Variable | P value |
| --- | --- |
|  |  |
| Sex | 0.84 |
| Age | `0.16 |
| BMI | 0.62 |
| ICS | 0.71 |
| OCS | 0.26 |
| **Overall** | 0.52 |
|  |  |

P-value for different covariates in a multivariate, multinomial logistic regression analysis with patient clusters (a nominal variable) as the outcome. BMI: body mass index, ICS: inhaled corticosteroids, OCS: oral corticosteroids.

Table E4. Selection of cytokines

| **Cellular source** | **Cytokine** |
| --- | --- |
| Th1 cell | IFN-γ, TNF |
| Th2 cell | IL-4, IL-5, IL-13 |
| Th17 cell | IL-17A, IL-17F, IL-22 |
| Treg cell | IL-10 |
| epithelium | IL-25, IL-1β, IL-6, TNF |
| macrophage | IL-1β, IL-6, TNF |

The selected cytokines are representative cytokines for Th1, Th2, Th17, Treg and epithelial cell activity with clear involvement in asthma pathogenesis at time of analysis. TSLP and IL-33 were analyzed in a previous cohort (11) but expression was close to the detection limit and only positive in respectively 1 and 3 out of 66 patients.

Figure legends

**Figure E1. CCC, pseudo F and t^2^ statistics to define number of patient clusters.**

The number of clusters is determined by taking the value after a steep increase of the CCC statistics, the highest value of the pseudo F statistics or the value before a steep increase (from right to left) of the pseudo T^2^. A five-cluster model was chosen for the dataset of all asthmatics (A). A six-cluster model was chosen for the dataset of steroid-naive asthmatics (B).

**Figure E2. Validation of patients’ cytokine profile in each cluster.**

Validation of the number of clusters and the patients’ cytokine profile in each cluster was performed by splitting the total cohort into 2 groups. Radar plots were generated by presenting the proportion of patients having a ‘cytokine-high’ mRNA expression profile as a set of points plotted along a set of axes radiating from a central point, and with the points connected by a line (Seys et al., Clin Exp Allergy, 2013). Upper row represents the cytokine profile of all 205 patients in each cluster (each column represents one cluster). Middle row (n=103) and lower row (n=102) represent cytokine profiles of respectively half of patients.

**Figure E3. Lung function characteristics of patient clusters after 3 years.**

FEV_1_ % predicted after 3 years are shown for asthmatics divided into 5 clusters: cluster 1: n=24, IL-5-high and IL-17F-high; cluster 2: n=15, IL-5-high and IL-17F-low; cluster 3: n=8, IL-6-high; cluster 25: n=15, IL-22-high; cluster 5: n=132. Data are represented as mean ± standard deviation. Data were analyzed by ANOVA and Dunn’s Multiple Comparison Test (^*^: p<0.05).

**Figure E4. Eosinophilic and neutrophilic airway inflammation among the different clusters.**

Schematic representation of eosionophilic and neutrophilic airway inflammation in asthmatic patients stratified by their sputum cytokine profile and cluster.
