# Supplementary figures and images for: Cluster analysis of sputum cytokine-high profiles reveals diversity in T(h)2-high asthma patients
Source: Respir Res. 2017 Feb 23;18:39. doi: 10.1186/s12931-017-0524-y (PMC5324270; doi:10.1186/s12931-017-0524-y)

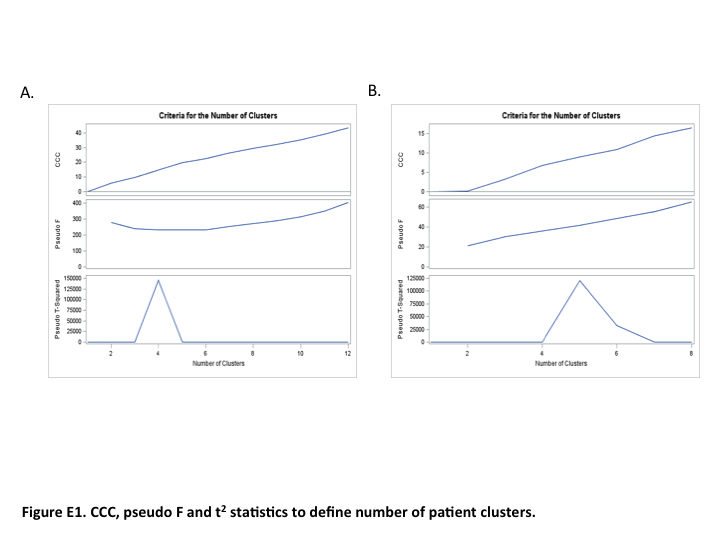

Supplement: Additional file 1: — Cluster analysis of sputum cytokine-high profiles reveals diversity in T(h)2-high asthma patients. (ZIP 247 kb) [file 12931_2017_524_MOESM1_ESM.zip › Figure E1.tif]

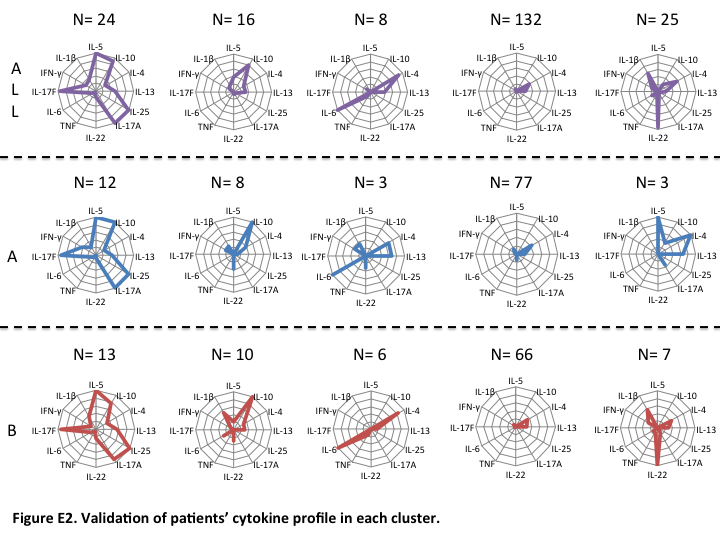

Supplement: Additional file 1: — Cluster analysis of sputum cytokine-high profiles reveals diversity in T(h)2-high asthma patients. (ZIP 247 kb) [file 12931_2017_524_MOESM1_ESM.zip › Figure E2.tif]

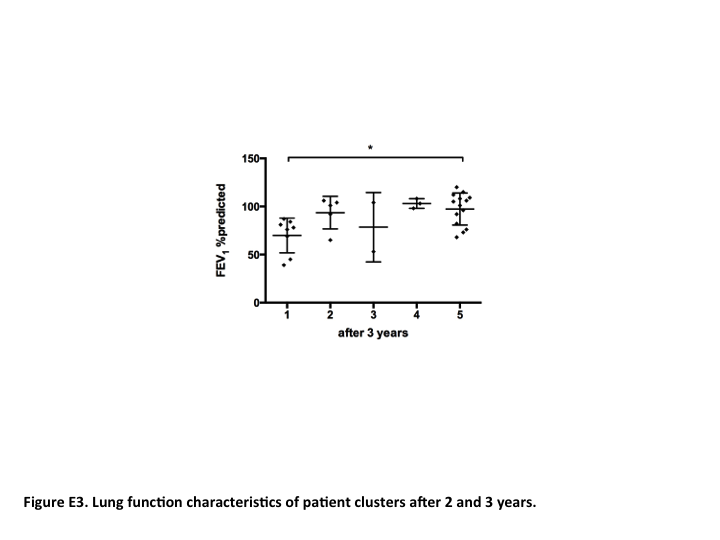

Supplement: Additional file 1: — Cluster analysis of sputum cytokine-high profiles reveals diversity in T(h)2-high asthma patients. (ZIP 247 kb) [file 12931_2017_524_MOESM1_ESM.zip › Figure E3.tif]

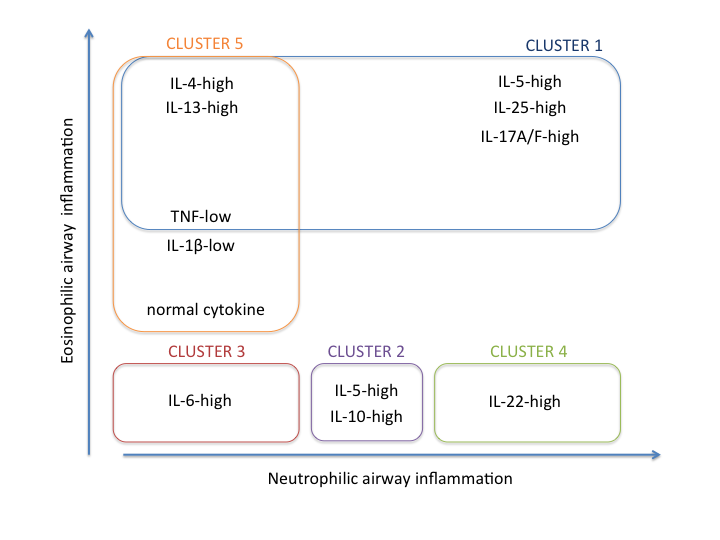

Supplement: Additional file 1: — Cluster analysis of sputum cytokine-high profiles reveals diversity in T(h)2-high asthma patients. (ZIP 247 kb) [file 12931_2017_524_MOESM1_ESM.zip › Figure E4.tif]
